# Changes in serum tumor markers in type 2 diabetes mellitus with microalbuminuria

**DOI:** 10.3389/fendo.2023.1247099

**Published:** 2023-12-07

**Authors:** Lina Chen, Shichun Du, Yan Bo Li, Qing Su, Jiangrong Zhang, Hongmei Zhang

**Affiliations:** ^1^ Department of Endocrinology, Xinhua Hospital Affiliated with Shanghai Jiaotong University School of Medicine, Shanghai, China; ^2^ Health Management Center, Xinhua Hospital Affiliated with Shanghai Jiaotong University School of Medicine, Shanghai, China

**Keywords:** tumor markers, microalbuminuria, type 2, diabetes mellitus, UACR

## Abstract

**Objectives:**

The objective of this study was to investigate changes in serum tumor markers in type 2 diabetes mellitus (T2DM) with microalbuminuria and analyze the relationship between tumor markers and microalbuminuria.

**Methods:**

A total of 956 T2DM patients aged 40–70 years hospitalized in the Department of Endocrinology, Xinhua Hospital, China, affiliated with Shanghai Jiaotong University School of Medicine, were enrolled from January 2018 to December 2020. The sample comprised 313 T2DM patients with microalbuminuria and 643 T2DM patients with normal urinary microalbumin levels. After assessing the changes in serum tumor markers in T2DM with microalbuminuria, we analyzed the risk of microalbuminuria by the serum tumor marker category using multiple logistic regression analysis.

**Results:**

Serum CEA, CA199, CA125, CA153, CA211, SCC, CA242, and CA50 levels were significantly higher in T2DM patients with microalbuminuria than in those without microalbuminuria, while serum AFP levels were lower in the microalbuminuria group (*P* < 0.05). Following adjustment of confounders, serum CEA, CA211, and SCC were independently associated with microalbuminuria in T2DM. An ROC curve was used to estimate the cutoff point of tumor markers for microalbuminuria. Taking the values under the cutoff points as a reference, values for CEA, CA211, and SCC above the cutoff points indicated a significantly high risk of microalbuminuria. The OR of increased CEA for microalbuminuria was 2.006 (95%CI 1.456–2.765), the OR of increased CA211 for microalbuminuria was 1.505 (95%CI 1.092–2.074), and the OR of increased SCC for microalbuminuria was 1.958 (95%CI 1.407–2.724).

**Conclusion:**

Several serum tumor markers were related to microalbuminuria in T2DM. Serum tumor markers such as CEA, SCC, and CA211 may indicate early diabetic nephropathy, particularly when elevated in combination.

## Introduction

Diabetes mellitus is a heterogeneous disease characterized by elevated blood glucose. Various genetic factors and environmental factors can lead to the dysfunction of pancreatic islet beta cells, resulting in a relative or absolute lack of insulin in the body, presenting as a hyperglycemic state (1). The International Diabetes Federation reported that global diabetes prevalence in adults aged 20–79 years reached 10.2% in 2021 and is projected to increase to 12.2% by 2045 (2). In 2021, global diabetes-related health costs were estimated to be 966 billion USD and are expected to reach 1054 billion USD by 2045 (2). China has witnessed one of the most dramatic rises in diabetes prevalence of anywhere in the world (3). It is well established that the prevalence of various cancers is higher in T2DM patients than in the general population (4). Serum tumor markers are widely used for cancer screening in clinical practice. Previous studies have reported connections between T2DM and several tumor markers are elevated in diabetic patients (5). Diabetic kidney disease (DKD) characterized by albuminuria is one of the most common vascular complications of diabetes. The urinary microalbumin-to-creatinine ratio (UACR) is usually recommended to screen and diagnose DKD. Whether a relationship exists between serum tumor markers and diabetic complications, particularly DKD, has rarely been reported. Hence, we conducted a cross-sectional study to explore the relationship between serum tumor markers and UACR and verify whether tumor markers are associated with microalbuminuria in T2DM. We also aimed to identify a new marker of early diabetic nephropathy and explain why tumor markers are increased in T2DM patients in the absence of malignant tumors.

## Methods

### Subjects

A total of 956 adult T2DM patients without a history of malignant tumor hospitalized in the Department of Endocrinology, Xinhua Hospital, China, affiliated with Shanghai Jiaotong University School of Medicine, were enrolled from January 2018 to December 2020. The sample comprised 313 T2DM patients with microalbuminuria and 643 T2DM patients with normal urinary microalbumin levels. The enrolled subjects were all hospitalized patients who signed the informed consent form for hospitalization when they were admitted and agreed that all their data during hospitalization could be used for future scientific research by our hospital. Extra informed consent was waived by the hospital ethics committee as the research was about hospitalized patients. Patients with a history of kidney disease, including chronic glomerulonephritis, acute nephritis, or urinary tract infection, were excluded, as were those with acute infection and autoimmune disease or those who received a malignant tumor diagnosis while hospitalized.

### Anthropometric and biochemical measurements

Anthropometric measurements, including height, weight, and blood pressure, were collected by medical staff. Other data collected included fasting blood glucose (FPG), 2-hour postprandial blood glucose (2hPG), glycosylated hemoglobin (HbA_1c_), fasting C-peptide (FC-P), 2-hour postprandial C-peptide (2hC-P), fasting insulin (FINS), 2-hour postprandial insulin (2hINS), liver function indexes, kidney function indexes, lipids profiles, tumor markers, and urinary microalbumin-to-creatinine ratio. We used the formulas to calculate the index as follows: BMI was calculated with the formula: weight (kg)/square of height (m²). HOMA-IR was calculated with the formula: FPG (mmol/L) * FINS (μU/mL)/22.5. Serum tumor markers were determined by the immunoassay method (cobas e 801 analyzer, Roche), while blood glucose, blood lipids, hepatic function, and renal function were determined by an automatic biochemical analyzer (Hitachi LABOSPECT 008 AS, Japan). Blood C-peptide and insulin levels were measured with the chemiluminescence method (BECKMAN COULTER UniCeL Dxl 800 Access immunoassay system, USA). Glycated hemoglobin was determined using high-performance liquid chromatography (Bio-Rad Variant II Turbo, USA). Urine microalbumin was measured by immunoturbidimetry (Siemens automatic protein analyzer, Germany).

### Statistical analysis

SPSS 22.0 software (SPSS Inc., Chicago, IL) was used to analyze the data. The data were expressed as mean± standard deviation or median with interquartile range. The comparison between the two groups was performed using the Mann–Whitney U test or independent samples t-test. Spearman correlation analysis and multiple stepwise regression analysis were used to estimate the associations of tumor markers with other variables. Binary logistic regression models were adopted to evaluate the odds ratios (ORs) for microalbuminuria. P values < 0.05 were considered statistically significant. An ROC curve was used to estimate the cutoff point of tumor markers for microalbuminuria.

## Results

### Clinical characteristics of the two groups

**Table 1** shows the basic clinical characteristics of the two groups. There was no difference in sex proportion, ALT, AST, LDL-C, FC-P, 2hC-P,and 2hFINS between the two groups. Compared to the group without microalbuminuria, age, diabetes duration, blood pressure, and BMI, SCr, TC, TG, HbA_1c_, FPG, 2hPG, FINS, and HOMA-IR were all higher in patients with microalbuminuria. Estimated glomerular filtration rate (eGFR) and HDL-C were lower in patients with microalbuminuria (detailed in **Table 1**).

**Table 1:** Clinical characteristics in T2DM with and without microalbuminuria.

Variables	T2DM with microalbuminuria (n=313)	T2DM without microalbuminuria (n=643)	P
Age (y)	61.72 ± 6.20	60.23 ± 6.58	0.001
Male (%)	63%	63%	–
Diabetic duration (y)	11(7~19)	10(3~14)	<0.001
SBP (mmHg)	140(127~153)	131(120~143)	<0.001
DBP (mmHg)	84(76~90)	80(73~88)	<0.001
BMI (kg/m^2^)	25.06(23.12~26.87)	24.57(22.48~26.43)	0.014
ALT (U/L)	19(14~28)	19(14~31)	0.361
AST (U/L)	19(15~24)	20(16~25)	0.090
SCr (umol/L)	64(51~81)	60(50~71)	0.001
eGFR (ml/min^-1^*1.73m^2^)	101.41(78.89~123.06)	108.87(92.62~127.74)	<0.001
TC (mmol/L)	4.47(3.82~5.23)	4.36(3.73~5.02)	0.037
TG (mmol/L)	1.86(1.27~2.77)	1.51(1.05~2.19)	<0.001
HDL-C (mmol L)	1.11(0.90~1.34)	1.14(0.98~1.37)	0.008
LDL-C (mmol/L)	2.76(2.17~3.37)	2.65(2.06~3.28)	0.118
HbA_1c_ (%)	8.60(7.50~10.20)	8.20(7.00~9.65)	0.002
FPG (mmol/L)	7.43(6.02~9.62)	7.21(5.74~8.90)	0.012
2hPG (mmol/L)	12.91(10.17~15.31)	12.11(9.24~15.08)	0.008
FC-P (nmol/L)	0.63(0.42~0.93)	0.60(0.41~0.82)	0.099
2hC-P (nmol/L)	1.40(0.98~2.20)	1.57(0.94~2.23)	0.213
FINS (pmol/L)	71.85(46.66~110.57)	63.09(41.75~90.29)	<0.001
2hINS (pmol/L)	245.05(182.26~404.14)	247.11(157.53~381.26)	0.176
HOMA-IR	4.02(2.56~7.16)	3.45(2.01~5.25)	<0.001

SBP, systolic blood pressure; DBP, diastolic blood pressure; BMI, body mass index; ALT, alanine aminotransferase; AST, aspartate aminotransferase; SCr, serum creatinine; eGFR, estimated glomerular filtration rate; TC, total cholesterol; TG, triglyceride; HDL-C, high-density lipoprotein cholesterol; LDL-C, low-density lipoprotein cholesterol; HbA_1c_, glycosylated hemoglobin; FPG, fasting blood glucose; 2hPG 2h postprandial glucose; FC-P fasting C-peptide; 2hC-P, 2h postprandial C-peptide; FINS, fasting insulin; 2hINS, 2h postprandial insulin; HOMA-IR, homeostasis model assessment index.

### Comparison of tumor markers between the two groups

Data were analyzed using the Mann–Whitney U test (**Table 2**). There was no statistically significant difference in CA724 and NSE between the two groups. AFP was lower in patients with microalbuminuria (*P* = 0.033), while serum CEA, CA199, CA125, CA153, CA211, SCC, CA242, and CA50 were all higher (detailed in **Table 2**).

**Table 2:** Tumor markers in T2DM with and without microalbuminuria.

Variables	T2DM with microalbuminuria	T2DM without microalbuminuria	Z	P
AFP (ng/mL)	2.26(1.71~3.29)	2.35(1.80~3.28)	-2.138	0.033
CEA (ng/mL)	3.09(2.03~4.27)	2.36(1.77~3.51)	-4.650	<0.001
CA199 (U/mL)	15.50(10.40~24.69)	12.43(8.72~18.83)	-4.314	<0.001
CA125 (U/mL)	9.84(7.00~13.94)	9.20(7.04~12.02)	-2.586	0.010
CA153 (U/mL)	9.62(7.20~14.28)	9.18(6.60~13.40)	-2.790	0.005
CA724 (U/mL)	2.57(1.68~5.39)	2.78(1.82~5.09)	-0.829	0.407
CA211(ng/mL)	2.40(1.80~3.33)	1.99(1.53~2.67)	-4.853	<0.001
NSE (ng/mL)	15.04(12.4~17.73)	15.60(12.73~18.67)	-1.263	0.207
SCC (ng/mL)	0.90(0.70~1.30)	0.70(0.50~1.00)	-5.439	<0.001
CA242 (U/mL)	6.38(4.20~10.15)	5.35(3.66~8.41)	-2.470	0.014
CA50 (U/mL)	9.87(6.20~15.70)	7.49(4.80~11.75)	-4.742	<0.001

AFP, Alpha-fetoprotein; CEA, Carcinoembryonic antigen; CA199, Carbohydrate antigen 199; CA125, Carbohydrate antigen 125; CA153, Carbohydrate antigen 153; CA724, Carbohydrate antigen 724; CA211, Carbohydrate antigen 211; NSE, Neuron-specific enolase; SCC, Squamous cell carcinoma antigen; CA242, Carbohydrate antigen 242; CA50, Carbohydrate antigen50.

### Variables independently associated with microalbuminuria

As shown in **Table 3**, following adjustment for all confounders, variables independently associated with microalbuminuria in T2DM patients were duration of T2DM (OR 1.043, 95%CI 1.020–1.066), SBP (OR 1.021, 95%CI 1.013–1.030), HbA_1c_ (OR 1.090, 95%CI 1.003–1.184), BMI (OR 1.053, 95%CI 1.002–1.106), TC (OR 1.221, 95%CI 1.059–1.409), TG (OR 1.220, 95%CI 1.101–1.351), HDL-C (OR 0.524, 95%CI 0.304–0.906), LDL-C (OR 1.218, 95%CI, 1.024–1.449), eGFR (OR 0.993, 95%CI 0.988–0.999), and HOMA-IR (OR 1.056, 95%CI 1.029–1.083). An ROC curve was used to estimate the cutoff point of tumor markers for microalbuminuria. Taking the values under the cutoff points as a reference, values of CEA, CA211, and SCC above the cutoff points indicated a significantly high risk of microalbuminuria. The OR of increased CEA for microalbuminuria was 2.006 (95%CI 1.456–2.765), the OR of increased CA211 for microalbuminuria was 1.505 (95%CI 1.092–2.074), and the OR of increased SCC for microalbuminuria was 1.958 (95%CI 1.407–2.724) (see **Table 4**).

**Table 3:** Variables independently associated with microalbuminuria by logistic analysis.

Variables	β	Exp (β) (95% CI)	P
Diabetic duration (y)	0.042	1.043 (1.020–1.066)	<0.001
SBP (mmHg)	0.021	1.021 (1.013–1.030)	<0.001
HbA_1c_ (%)	0.086	1.090 (1.003–1.184)	0.043
BMI (kg/m^2^)	0.052	1.053 (1.002–1.106)	0.040
eGFR (ml/min^-1^*1.73m^2^)	-0.007	0.993 (0.988–0.999)	0.015
TC (mmol/L)	0.200	1.221 (1.059–1.409)	0.006
TG (mmol/L)	0.198	1.220 (1.101–1.351)	<0.001
HDL-C (mmol/L)	-0.646	0.524 (0.304–0.906)	0.021
LDL-C (mmol/L)	0.197	1.218 (1.024–1.449)	<0.001
CEA (ng/mL)	0.100	1.172 (1.063–1.293)	0.034
CA211 (ng/mL)	0.263	1.301 (1.123–1.508)	<0.001
SCC (ng/mL)	0.266	1.304 (1.035–1.430)	0.024
HOMA-IR	0.054	1.056(1.029–1.083)	<0.001

**Table 4:** Adjusted ORs and 95% CIs for microalbuminuria according to tumor marker categories.

	Adjusted OR (95%CI)		
Variables	Crude OR(95%CI)	Model 1	Model 2
CEA< 2.635 (ng/mL)	1	1	1
CEA≥2.635 (ng/mL)	2.023** (1.532–2.670)	1.975** (1.483–2.629)	2.006** (1.456–2.765)
CA211<2.195 (ng/mL)	1	1	1
CA211≥2.195 (ng/mL)	1.783** (1.354–2.349)	1.673** (1.265–2.213)	1.505* (1.092–2.074)
SCC< 0.750 (ng/mL)	1	1	1
SCC≥0.750 (ng/mL)	2.208** (1.669–2.920)	2.142** (1.609–2.852)	1.958** (1.407–2.724)

Model 1 adjusted for age and sex.

Model 2 further adjusted for age, sex, diabetic duration, blood glucose, HbA_1c_, BP, BMI, lipid profiles, and FC-P.*p< 0.05, **p < 0.001.

### Variables independently related to CEA, CA211, and SCC

As detailed in **Table 5**, variables independently associated with CEA were HbA_1c_, LDL-C, age, and sex. Variables independently associated with CA211 were HbA_1c_, LDL-C, age, diabetes duration, SCr, DBP, and FC-P. Variables independently associated with SCC were SCr, age, and FC-P.

**Table 5:** Variables independently related to CEA, CA211, and SCC by multiple linear regression analysis.

	Variables	β	t	P
CEA (ng/mL)	HbA_1c_ (%)	0.228	6.946	<0.001
	LDL-C (mmol/L)	0.077	2.290	0.022
	Age (y)	0.114	3.403	<0.001
	Sex	-0.233	-6.977	<0.001
CA211 (ng/mL)	DBP (mmHg)	0.109	3.320	<0.001
	FC-P (nmol/L)	0.109	3.157	0.002
	Age (y)	0.101	2.954	0.003
	Duration(y)	0.070	2.039	0.042
	HbA_1c_ (%)	0.101	3.028	0.003
	LDL-C (mmol/L)	0.077	2.290	0.022
	SCr (umol/L)	0.169	4.993	<0.001
SCC (ng/mL)	FC-P (nmol/L)	0.082	2.369	0.018
	Age (y)	0.078	2.292	0.022
	SCr (umol/L)	0.158	4.514	<0.001

## Discussion

China is among the countries with the highest prevalence of diabetes worldwide, with the number of patients estimated to exceed 140 million in 2021 and projected to reach over 174 million by 2045 (2). As reported by Zheng et al., diabetes mellitus is the ninth leading cause of mortality globally (6). A previous study that investigated the relationship between T2DM and malignant tumors observed a significant correlation (7). Chronic complications of T2DM lead to increased mortality and morbidity and severely affect the life expectancy and quality of life of patients. DKD is one of the most common vascular complications of diabetes. Clinically, the stage of diabetic nephropathy is primarily determined based on the urinary albumin excretion rate, glomerular filtration rate, creatinine, and total urinary protein. The American Diabetes Association screens and diagnoses DKD according to UACR, defining UACR < 30μg/mg, 30-299μg/mg, and > 300μg/mg as normal, microalbuminuria, and macroalbuminuria, respectively. Gerstein et al. showed that any degree of albuminuria is a risk factor for cardiovascular events in individuals with T2DM and that risk increases progressively with UACR elevation (8). A previous study found that CA153 was negatively related to eGFR and positively related to HbA_1c_ and FPG in patients with T2DM (9). Turgutalp et al. found that the urinary protein excretion rate was correlated with CA125, CA153, and CA199 (10). So far, numerous studies have shown that some serum tumor markers are higher in patients with T2DM than in healthy individuals, but few studies have investigated the relationship between serum tumor markers and microalbuminuria in T2DM patients.

Our present study revealed that tumor markers were related to microalbuminuria in T2DM. Most of the serum tumor markers, namely, CEA, CA199, CA125, CA153, CA211, SCC, CA242, and CA50, were increased in the T2DM with microalbuminuria group, while serum AFP was decreased. CA199 has high sensitivity in the diagnosis of pancreatic cancer and, as such, is regarded as the best validated biomarker of pancreatic cancer (11). T2DM is associated with a status of chronic inflammation; pancreatic inflammation leads to impairment of pancreatic exocrine gland function, resulting in increased CA199 levels (12). A previous study indicated that CA199 levels in T2DM patients with microvascular complications including neuropathy, diabetic nephropathy, and retinopathy were significantly increased in comparison with those without microvascular complications (13). It has also been reported that renal impairment could lead to increased serum CA125 levels, which have been independently correlated with urinary microalbumin (14). Our results are in agreement with this finding. Furthermore, as our studied subjects were T2DM patients, the levels of CA125 were significantly higher in T2DM patients with microalbuminuria than in those without microalbuminuria. In recent years it has been well established that inflammation promotes the occurrence and progression of T2DM, and the activated inflammatory status may play an important role in the occurrence and progression of DKD (15, 16). DKD is an inflammatory disease with increased serum high-sensitivity C-reactive protein levels (17).

CA153 is a glycoprotein that has a proven association with a wide range of cancers (18). CA153 is a product of the Mucin 1 (MUC1) gene, a transmembrane protein expressed on the surface of most epithelial cells (19, 20). Some inflammatory factors such as tumor necrosis factor (TNF), interleukin-1 (IL-1), and interleukin-6 (IL-6) can promote the expression of MUC1 (21), so in an inflammatory state, serum CA153 may be increased. Accordingly, our results showed serum CA153 levels were higher in T2DM patients with microalbuminuria than in those without microalbuminuria. Other researchers have found that serum CA153 levels were negatively correlated with eGFR in diabetic patients (9). CA50, initially reported as a specific antigen expressed on the surface of colorectal cancer cells (22), has also been discovered in other malignant tumors, including lung cancer, pancreatic cancer, liver cancer, gastric cancer, uterine cancer, and bladder cancer (22). Nevertheless, it has been observed to be increased in some benign diseases and notably in patients who suffer from T2DM and pancreatitis. In such cases, the level of serum CA50 decreases after remission of inflammation (22). We suppose that inflammation may be the underlying reason for increased serum CA50 among T2DM patients with microalbuminuria. Our study found that the level of AFP was markedly lower in T2DM patients with microalbuminuria than in patients without microalbuminuria, which is consistent with Turgutalp’s conclusions. Turgutalp et al. (10) found that urinary protein excretion was correlated with tumor markers. The possible reasons for the decrease in AFP are loss from urine, increased catabolic rate, decreased synthesis rate, changes in molecular structure, and usage of drugs.

Following adjustment for all confounders, the tumor markers independently associated with microalbuminuria in T2DM patients were CEA, CA211, and SCC. The increased serum CEA levels in T2DM patients with microalbuminuria in our study are consistent with the conclusion of previous research (23). As DKD is more common in patients with poor glycemic control, in the long-term, serum CEA level would be expected to significantly increase due to glucotoxicity damage to the digestive tract. Long-term hyperglycemia could lead to raised levels of advanced glycation end products, resulting in vascular endothelial dysfunction and oxidative stress (23). Another study demonstrated that there exists a positive relationship between serum CEA levels and leukocyte counts in adults; as such, elevated serum CEA levels may reflect a chronic state of inflammation (24). Diabetes mellitus and DKD are chronic inflammatory diseases.

Another reason for increased CEA levels in microalbuminuria in T2DM may be because of the glomerular ultrafiltrate function. According to a previous study, plasma proteins with molecular masses higher than albumin are mostly restricted from passing into the glomerular ultrafiltrate, and thus, only a small proportion can be detected in urine (25). CEA is one of several proteins of this type. In our study, serum levels of SCC and CA211 were also independently associated with microalbuminuria in T2DM. The specific mechanism remains unclear, and we did not find any relevant studies reporting on the relationship between SCC, CA211, and microalbuminuria. The possible reasons may include inflammation, oxidative stress, imbalance of synthesis, catabolism and excretion, and other related factors.

In conclusion, compared to T2DM patients without microalbuminuria, T2DM patients with microalbuminuria were found to have higher levels of CEA, CA199, CA125, CA153, CA211, SCC, CA242, and CA50. Serum CEA, CA211, and SCC were independently correlated with microalbuminuria in T2DM. When serum tumor markers are elevated in diabetic patients, especially when they are increased in combination, it is necessary to screen for diabetic nephropathy in addition to malignant tumors.

Our research has some advantages and limitations. Firstly, the studied population was relatively large. Second, the items of tumor indicators were completely detected, and almost all of the tumor markers were analyzed. Third, our study was the first study to report the relationship between all of the tumor markers and DKD. The limitation of the current study is that it was cross-sectional, and so we cannot ascertain the causality of serum tumor markers for the risk of DKD. As such, further studies are warranted.

## Data availability statement

The datasets presented in this study can be found in online repositories. The names of the repository/repositories and accession number(s) can be found in the article/supplementary material.

## Ethics statement

The studies involving humans were approved by Ethics Committee of Xinhua Hospital Affiliated to Shanghai Jiaotong University School of Medicine. The studies were conducted in accordance with the local legislation and institutional requirements. The enrolled subjects are all hospitalized patients who have signed the informed consent form for hospitalization when they were admitted, and have agreed that all their data during hospitalization could be used for future scientific research by our hospital. Extra informed consent form was waived by the hospital ethics committee if the research is about hospitalized patients.

## Author contributions

HZ designed the study. LC and SD performed the statistical analysis and drafted the manuscript with assistance from HZ. JZ and LC collected the data. QS and JZ contributed to the specification of the analyses and critically reviewed and edited the manuscript. HZ and JZ are the guarantors of this work, had full access to all the data in the study, and take responsibility for the integrity of the data and the accuracy of the data analysis. All authors contributed to the article and approved the submitted version.
